# Aortocaval Fistula in a Behcet's Disease Patient

**DOI:** 10.1155/2009/536424

**Published:** 2009-12-20

**Authors:** Yusuf Ata, Tamer Turk, Mihriban Demir, Filiz Ata, Senol Yavuz

**Affiliations:** ^1^Department of Cardiovascular Surgery, Bursa Yuksek Ihtisas Education and Research Hospital, 16330 Bursa, Turkey; ^2^Department of Anesthesiology, Bursa Yuksek Ihtisas Education and Research Hospital, 16330 Bursa, Turkey

## Abstract

Behcet's disease (BD) is a chronic, recurrent, systemic disease that is characterized by oral and genital ulcers and oculocutaneous inflammatory lesions. Cardiovascular involvement especially large artery involvement is a serious and vital complication of BD. Pseudoaneurysms in the major arteries may be the cause of sudden death in BD. In our case a pulsatile abdominal mass was determined to be an aortic pseudoaneurysm associated with BD and an aortocaval fistula. Here we report this case and a short review of literature because this is the first reported aortocaval fistula in a BD patient in English literature.

## 1. Background

Behcet's disease (BD) is a well-known autoimmune multisystemic disorder that is characterized by oral and genital ulcers and oculocutaneous inflammatory lesions. Cardiovascular, pulmonary, neurological, articular, and gastrointestinal involvements are common features. The most important predictor of morbidity and mortality is the vascular complications especially arterial involvement of this disease [1, 2]. The typical vascular presentations are both arterial occlusive and aneurysm formation in the medium and large vessels. The inflammatory obliteration of the vasa vasorum of the arterial wall may lead to perforation or pseudoaneurysm formation. 

Fistula formation to the inferior vena cava is a rare complication of abdominal aorta aneurysm (AAA). The incidence of aortocaval fistulae (ACF) varies between 0.2% and 0.9% as a complication of AAA [3]. Spontaneous erosion of the aneurysm into the vena cava is the major cause of the primary ACF. Most of these aneurysms are atherosclerotic in nature. 

We report a rare case of ACF and abdominal aortic pseudoaneurysm due to BD in a young male.

## 2. Case Report

A 24-years-old man referred to our hospital with a diagnosis of an AAA. He also had a diagnosis of BD which was established in another hospital because of a history of uveitis, recurrent oral, and genital ulcerations. With these findings our patient fulfilled the international criteria for diagnosis of BD (Table 1) [4]. An informed consent is obtained from the patient.

**Figure 1:**
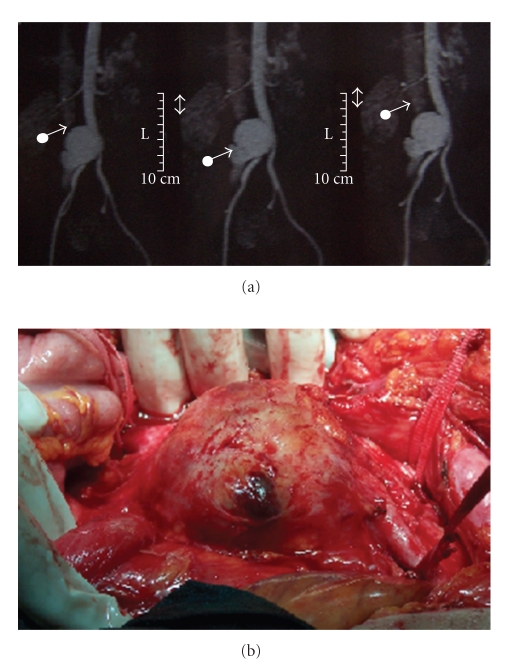
(a) MRI of abdominal aortic pseudoaneurysm showing a small amount of aortacaval connection. (b) Operative image of pseudoaneurysm.

The patient had a complaint of abdominal pain. Physical examination revealed a pulsatile abdominal mass without abdominal bruit. Routine laboratory tests were normal except elevated erythrocyte sedimentation rate and high C-reactive protein levels. MRI revealed a 6 × 8 cm pseudoaneurysm at the distal abdominal aorta just proximal to the bifurcation of the aorta associated with an ACF (Figures 1(a)-1(b)).

**Figure 2:**
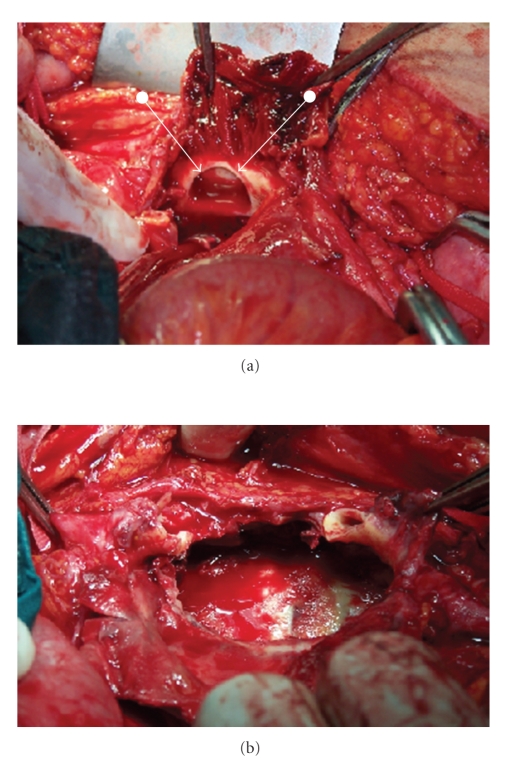
(a) Pseudoaneurysm after opening the aneurysmal sac and showing the neck of the pseudoaneurysm at the abdominal aorta. (b) Huge pseudoaneurysm after evacuating the thrombus and resecting the diseased part of the abdominal aorta.

After medical treatment the patient was explored with midline laparatomy. The abdominal aorta was explored from distal of the renal arteries. Aorta and the common iliac arteries are clamped after heparin administration. There was a huge pseudoaneurysm nearly to rupture with a 1–1.5 cm neck (Figure 2(a)). The real size of the pseudoaneurysm was bigger than MRI image and was surrounding the aorta including the retroaortic region (Figure 2(b)). After opening the pseudoaneurysm pouch and evacuating the thrombus we found the venous bleeding into the aneurysm pouch at the right and distal part of the pseudoaneurysm that was coming from a 0.5 cm connection from the inferior vena cavae. The fistula was sutured primarily by manual compression of distal and proximal cavae. After reaching to the disease free and normal segments of the aorta we performed a Dacron greft interposition to the abdominal aorta using teflon felt at the anastomoses. The patient had an uneventful postoperative course and was discharged at the seventh postoperative day.

## 3. Discussion

BD is an autoimmune vasculitis seen most often in young adults of Mediterranean or Far East region. It was first described by a Turkish dermatologist, Hulusi Behcet, in 1937 [5]. It is characterized with the triad of recurrent oral aphthous ulcers, genital lesions, and uveitis.

Arterial involvement is a rare but a serious complication of BD that leads to mortality and morbidity [2, 6]. Arterial involvement may be occlusive or aneurysmal in nature. Aneurysms usually involve medium and large-sized arteries such as thoracic aorta, abdominal aorta, pulmonary, carotid, subclavian, and femoral arteries [6–9]. Aneurysmal degeneration is thought to occur because of obliterative endarteritis of the vasa vasorum of the associated artery [6, 10]. Obliterative endarteritis of the vasa vasorum causes destruction of the medial layer and fibrosis. These arterial wall changes may lead to formation of a true aneurysm or produce a pseudoaneurysm due to arterial wall perforation [6, 10]. Rupture of the pseudoaneurysms located at areas that are difficult to access and that can bleed into body cavities, like the aorta, pulmonary, and subclavian arteries, is the major causes of mortality [11, 12]. 

The formation of an ACF between the abdominal aorta and the inferior vena cava is rare but a fatal complication of AAAs that is observed in less than 1% of all AAAs and 3-4% of ruptured AAAs [13]. In most of the cases, the rupture is spontaneous and fistulizing AAAs is atherosclerotic but rarely has been reported to be in association with mycotic, sphylitic, and other aneurysm forming diseases such as Ehlers-Dunlos syndrome, Marfan's syndrome, and Takayasu arteritis. As far as our review there is no reported ACF due to pseudoaneurysm of the abdominal aorta associated with BD in the literature. 

Preoperative diagnosis of ACF is sometimes difficult because the classical triad of abdominal pain and pulsatile abdominal mass with machinery-like bruit and high-output cardiac failure are present only in 20%–50% of all cases [14, 15]. Possible reasons for not making a preoperative diagnosis are thought to be because of a shock that is obscuring symptoms when an emergent operation is needed and decreased shunt through ACF due to compression of the inferior vena cavae by a large aneurysm and partial occlusion of the ACF due to mural thrombus which leads to decreased shunt [15, 16]. We did not note abdominal bruit in our patient due to mural thrombus in the aneurysmal sac causing partial obstruction and partial compression of the inferior vena cavae.

A definitive preoperative diagnosis offers advantages in surgical management of ACF. Computerized tomography (CT) is the usual way of evaluating abdominal aortic aneurysm. Ultrasonographic scanning, Magnetic Resonance Imagination (MRI), and angiography are also used for diagnosis [15–17]. In nearly half of the cases diagnosis is established during the surgical procedure [3].

Surgical repair is required in case of an AAA and ACF. Challenging problem during surgery is the venous bleeding from the fistula. This may be managed by a simple maneuver; distal and proximal manual compression of the vena cavae or transfemoral insertion of an occluding balloon catheter. Surgical mortality of the repair of ACF ranges from 10% to 66% [3, 14, 16]. Recently, endovascular treatment of AAAs has gained popularity with promising results especially in patients associated with BD [11, 18].

According to our knowledge, our case is the first abdominal aortic pseudoaneurysm associated with ACF in a BD patient in English literature. 

As a conclusion, diagnosis of BD should always be considered in patients who present with aortic aneurysm or pseudoaneurysm especially in young adults from Mediterranean region or Asia. Additionally, ACF associating with AAA or pseudoaneurysm should be kept in mind as a complication. Patients with BD should also be under regular evaluation for diagnosis of arterial complications. Surgery or endovascular treatment modalities must be considered for any aneurysm or pseudoaneurysm complicating BD with a risk of rupture or ACF formation. 

## Figures and Tables

**Table 1:** International study group criteria for the diagnosis of Behçet's disease.

Major criteria	Recurrent oral ulceration	Minor aphthous, major aphthous, or herpetiform ulceration
		observed by physician or patient that recurred at least three times in one 12-month period
		
	Recurrent genital ulceration	Aphthous or scarring observed by physician or patient
	Eye lesions	Anterior or posterior uveitis or cells in vitreous on slit lamp
Plus two of		examination; or retinal vasculitis observed by ophthalmologist
Minor criteria	Skin lesions	Erythema nodosum observed by physician or patient,
		pseudofolliculitis, papulopustular or acneiform nodules (observed by the physician in a postadolescent patient, not receiving corticosteroid treatment)
	Positive pathergy test	Read by a physician at 24–48 hours, performed with oblique
		insertion of a 20-gauge or smaller needle under sterileconditions
